# Strain-resolved metagenomic analysis of the gut as a reservoir for bloodstream infection pathogens among premature infants in Singapore

**DOI:** 10.1186/s13099-023-00583-8

**Published:** 2023-11-16

**Authors:** Sarah M. Heston, Charis Shu En Lim, Chengsi Ong, Mei Chien Chua, Matthew S. Kelly, Kee Thai Yeo

**Affiliations:** 1 Division of Pediatric Infectious Diseases, Duke University School of Medicine, Durham, NC USA; 2 Department of Neonatology, KK Women’s and Children’s Hospital, Singapore, Singapore; 3 Duke-NUS Medical School, Singapore, Singapore; 4 Department of Nutrition and Dietetics, KK Women’s and Children’s Hospital, Singapore, Singapore

**Keywords:** Preterm neonates, Early-onset sepsis, Late-onset sepsis, Intestinal microbiome, inStrain

## Abstract

**Background:**

Gut dysbiosis contributes to the high risk of bloodstream infection (BSI) among premature infants. Most prior studies of the premature infant gut microbiota were conducted in Western countries and prior to development of current tools for strain-resolved analysis.

**Methods:**

We performed metagenomic sequencing of weekly fecal samples from 75 premature infants at a single hospital in Singapore. We evaluated associations between clinical factors and gut microbiota composition using PERMANOVA and mixed effects linear regression. We used inStrain to perform strain-level analyses evaluating for gut colonization by BSI-causing strains.

**Results:**

Median (interquartile range) gestation was 27 (25, 29) weeks, and 63% of infants were born via Cesarean section. Antibiotic exposures (PERMANOVA; R^2^ = 0.017, p = 0.001) and postnatal age (R^2^ = 0.015, p = 0.001) accounted for the largest amount of variability in gut microbiota composition. Increasing postnatal age was associated with higher relative abundances of several common pathogens (*Enterococcus faecalis*: p < 0.0001; *Escherichia coli:* p < 0.0001; *Klebsiella aerogenes:* p < 0.0001; *Klebsiella pneumoniae:* p < 0.0001). Antibiotic exposures were generally associated with lower relative abundances of both frequently beneficial bacteria (e.g., *Bifidobacterium* species) and common enteric pathogens (e.g., *Enterobacter*, *Klebsiella* species). We identified strains identical to the blood culture isolate in fecal samples from 12 of 16 (75%) infants who developed BSI, including all infections caused by typical enteric bacteria.

**Conclusions:**

Antibiotic exposures were the dominant modifiable factor affecting gut microbiota composition in a large cohort of premature infants from South-East Asia. Strain-resolved analyses indicate that the gut is an important reservoir for organisms causing BSI among premature infants.

**Supplementary Information:**

The online version contains supplementary material available at 10.1186/s13099-023-00583-8.

## Background

Sepsis remains a major cause of mortality and morbidity among premature infants. Early-onset sepsis (EOS; onset ≤ 72 h of birth) occurs in 1–2% of very low birth weight (VLBW) infants and is associated with a case-fatality rate of approximately 20% [1–3]. Late-onset sepsis (LOS; onset > 72 h after birth) affects up to 24% of VLBW infants and is associated with a 30% increase in mortality, with the risk of death being highly dependent on the causative pathogen [1, 4–6]. While EOS cases are believed to originate from direct acquisition of microorganisms during the birthing process, recent studies indicate that a large proportion of LOS cases may occur from translocation of microbes from the gut [7, 8]. Additionally, prior studies reported that LOS episodes among premature infants are often preceded by alterations of the gut microbiota, including losses of microbial diversity and commensal bacteria and high abundances of common enteric pathogens [8–15]. Recent evidence suggests that the altered gut microbiota of premature infants may promote a pro-inflammatory microenvironment that increases gut permeability and microbial translocation [16]. However, most studies of the premature infant gut microbiome were performed in the United States and Europe, with relatively little known regarding factors that impact the gut microbiome and precede LOS among premature infants in Asian settings [17–19]. Additionally, bioinformatic tools for measuring genetic heterogeneity between microbial populations have advanced substantially in recent years, providing the opportunity to study microbial strain dynamics within the premature infant gut with unparalleled resolution.

In this study, we analyze metagenomic sequencing data from 581 fecal samples collected from 75 premature infants cared for in a single neonatal intensive care unit (NICU) in Singapore. We identify patient and treatment factors that influence gut microbiome composition among infants cared for in this setting. Additionally, we use strain-resolved metagenomic analyses to describe the dynamics of gut colonization by microbial strains causing bloodstream infections (BSI) in this patient population.

## Results

### Description of the study population

We collected fecal samples up to twice weekly from birth through 65 days of age from 173 premature infants born at or before 30 weeks gestational age at the KK Women’s and Children’s Hospital (KKH) Neonatal Intensive Care Unit (NICU) between June 2019 and May 2021. The analyses presented herein were limited to 75 infants from whom once weekly fecal samples were selected for shotgun metagenomic sequencing. For the current analyses, we included all neonates who developed a BSI and a random sampling of the remaining infants who had available weekly fecal samples. Most (59%) infants in this study population were female and nearly two-thirds (63%) were born via Cesarean section (Table 1). Median (interquartile range [IQR]) gestational age and birthweight were 27 (25, 29) weeks and 955 (763, 1210) grams, respectively. The overwhelming majority (88%) of infants received one or more antibiotics during the study period, with aminoglycosides (88%) and penicillins (87%) being the most frequently administered antibiotic classes and gentamicin (85%) and benzylpenicillin (80%) being the most frequently prescribed antibiotics (Table 2). All infants received a single-strain probiotic (*Bifidobacterium breve* M16V) from the time that they started enteral feeding until 36 weeks adjusted gestational age. Four (5%) infants died prior to hospital discharge.

**Table 1:** Characteristics of the 75 premature infants included in the study population

Characteristics	*N* (%)
Gestational age (weeks), median (IQR)	27 (25, 29)
Birthweight (grams), median (IQR)	955 (763, 1210)
Sex	
Female	44 (59%)
Male	31 (41%)
Race	
Chinese	35 (47%)
Indian	6 (8%)
Malay	15 (20%)
Other	19 (25%)
Delivery mode	
Cesarean section	47 (63%)
Vaginal	28 (37%)
Enteral feeding during study period	
Breast milk only	45 (60%)
Formula only	0 (0%)
Both	30 (40%)
Outcomes	
Bloodstream infection	16 (21%)
Death	4 (5%)

*IQR* interquartile range

**Table 2:** Antibiotic exposures among the study population

Antibiotics	*N* (%)	Treatment days, median (IQR)
Aminoglycosides	65 (87%)	5 (3, 10)
Amikacin	36 (48%)	
Gentamicin	64 (85%)	
Carbapenems	18 (24%)	0 (0, 0)
Meropenem	18 (24%)	
Cephalosporins	27 (36%)	0 (0, 3)
Cefazolin	5 (7%)	
Cefepime	2 (3%)	
Cefotaxime	25 (33%)	
Ceftazidime	3 (4%)	
Glycopeptides	6 (8%)	0 (0, 0)
Vancomycin	6 (8%)	
Macrolides	2 (3%)	0 (0, 0)
Azithromycin	1 (1%)	
Erythromycin	1 (1%)	
Nitroimidazoles	11 (15%)	0 (0, 0)
Metronidazole	11 (15%)	
Penicillins	66 (88%)	6 (3, 11)
Amoxicillin	1 (1%)	
Ampicillin	7 (9%)	
Ampicillin-sulbactam	3 (4%)	
Benzylpenicillin	60 (80%)	
Cloxacillin	35 (47%)	

*IQR* interquartile range

### Gut microbiome composition is highly dynamic and shaped by antibiotic exposures

**Fig. 1:**
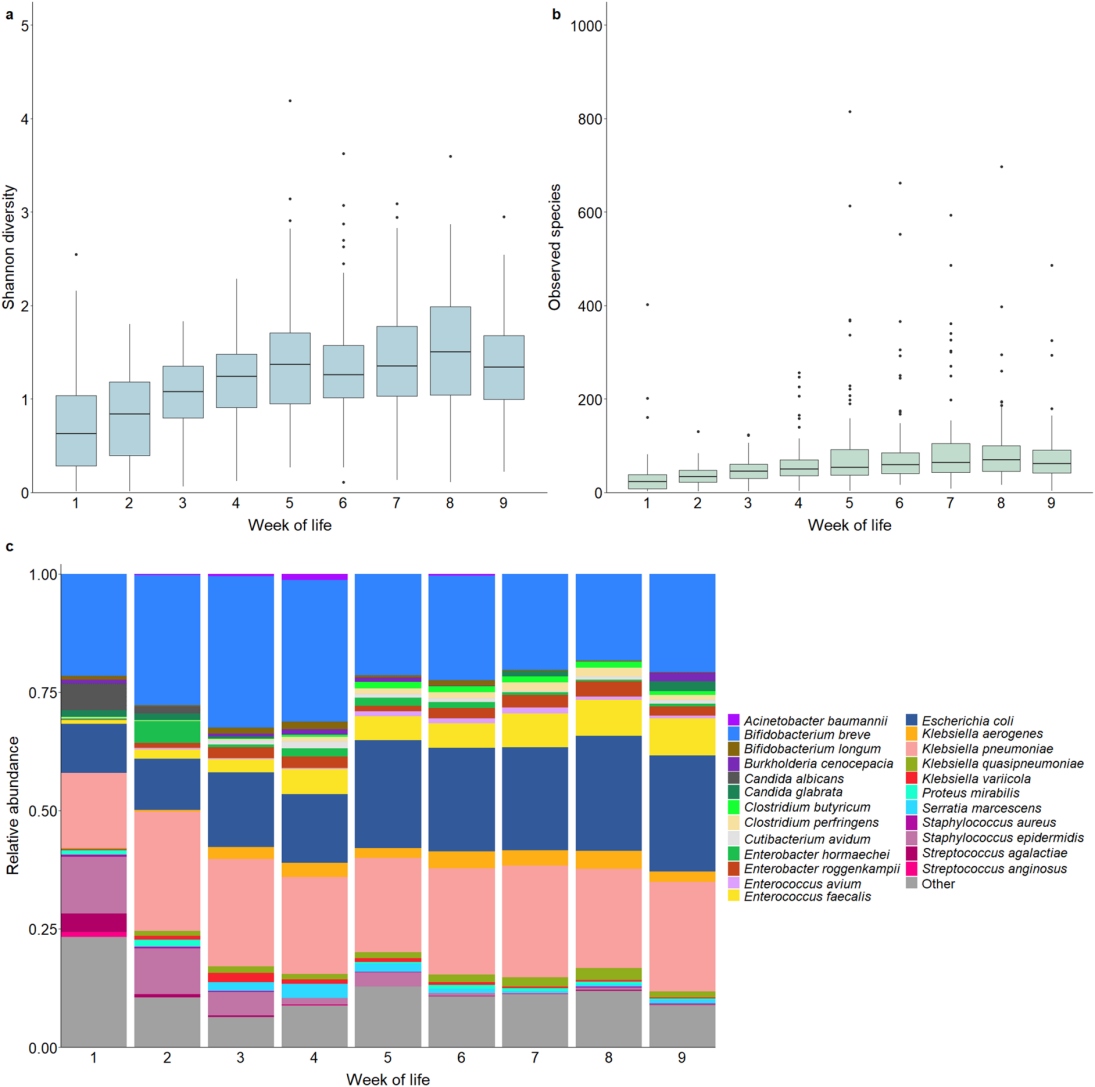
Premature infant gut microbiome alpha diversity and composition by postnatal age. Box and whisker plots of **a** the Shannon diversity index and **b** number of observed unique microbial species are shown by week of life. The horizontal lines represent the median value; boundaries of the rectangle correspond to the 25th and 75th percentiles; whiskers extend to values at 1.5 * the interquartile range; points represent outlier values. **c** Relative abundances of the 20 most highly abundant microbial species and BSI-causing species by postnatal age

We performed shotgun metagenomic sequencing of weekly fecal samples, with 581 fecal samples [median (IQR) of 8 (7, 9) samples per infant] passing quality control procedures. A total of 9,605,942,085 metagenomic reads were included in gut microbiome analyses, with a median (IQR) sequencing depth of 5.9 (4.4, 34.9) million reads per sample. Median (IQR) Shannon index and number of unique microbial species in these fecal samples were 1.17 (0.79, 1.53) and 48 (32, 72) respectively (Fig. 1a, b). Shannon diversity and the number of unique microbial species in fecal samples increased with increasing postnatal age (linear mixed effects models; Shannon index: β = 0.11, p < 0.0001; number of species: β = 1.18, p < 0.0001). A total of 3,830 species were identified in these samples (Fig. 1c), with the most highly abundant species being *B. breve* (mean relative abundance of 24%), *Klebsiella pneumoniae* (22%), *Escherichia coli* (18%), *Enterococcus faecalis* (5%), *Staphylococcus epidermidis* (4%), and *Klebsiella aerogenes* (2%).

**Fig. 2:**
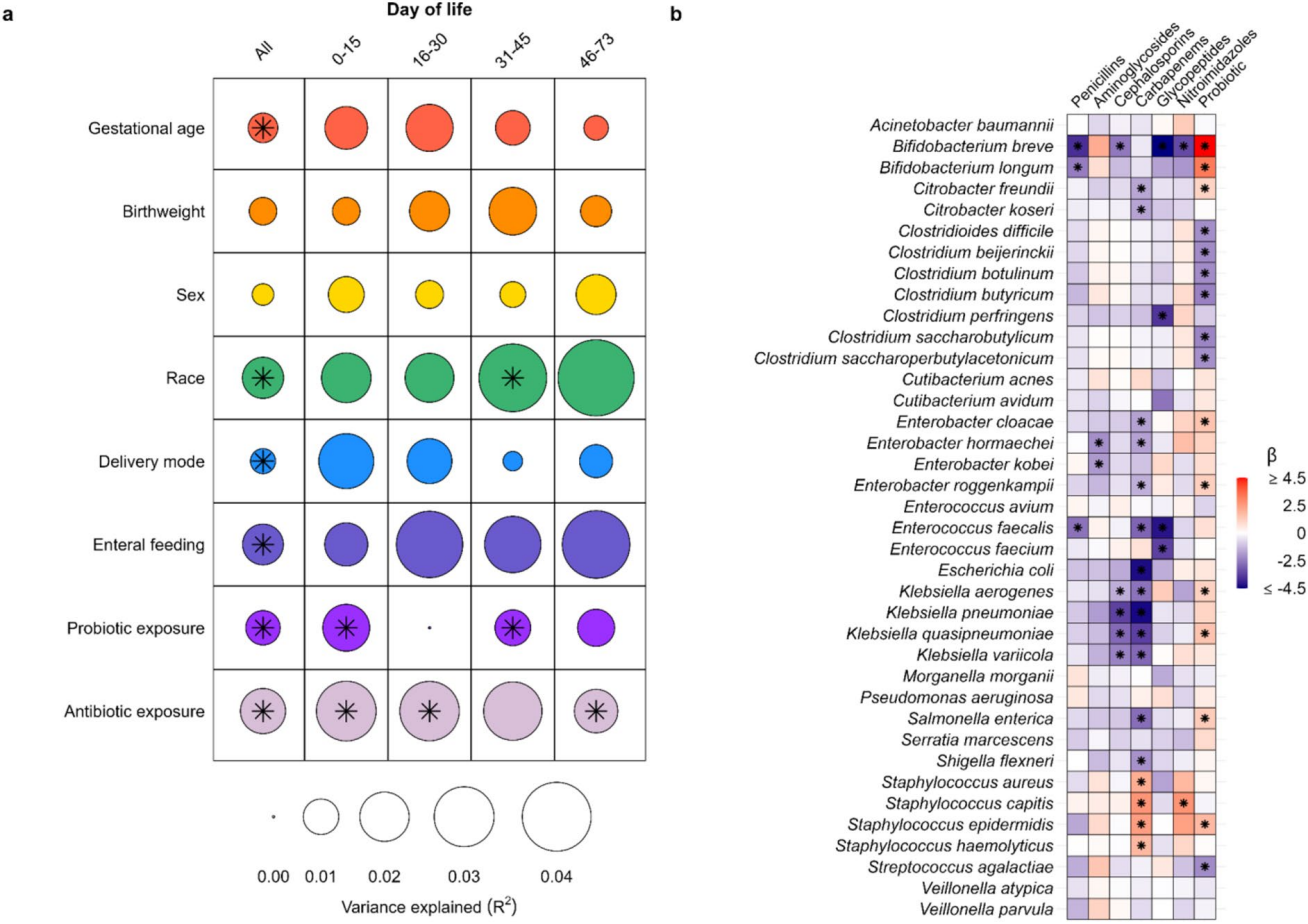
Associations between clinical factors and gut microbiome composition. **a** Bubble plot depicting the amount of variation in gut microbial composition explained by given clinical variables and as measured by PERMANOVA. The size of bubbles represents the amount of variance explained by the variable; asterisks indicate statistical significance (p < 0.05). The first column depicts results from analyses of all samples adjusting for postnatal age; subsequent columns show results from analyses stratified by postnatal age. **b** Heatmap displaying associations between antibiotic and probiotic exposures and the relative abundances of microbial species within the gut microbiota as estimated by mixed effects linear regression. Only microbial species with a minimum mean relative abundance of 0.01% and a sample prevalence of at least 10% are shown. Boxes are shaded based on the direction and size of the effect as determined from the model beta coefficients; asterisks indicate statistical significance (p < 0.05)

We next sought to identify patient and treatment factors that influenced gut microbiome development among the study population. We found that antibiotic exposures (PERMANOVA on Bray–Curtis dissimilarity; R^2^ = 0.017, p = 0.001) and postnatal age (R^2^ = 0.015, p = 0.001) accounted for the largest variability in gut microbiota composition (Fig. 2a). Other factors that were associated with gut microbiome composition included race (R^2^ = 0.014, p = 0.001), enteral feeding type (R^2^ = 0.013, p = 0.003), probiotic exposure (R^2^ = 0.009, p = 0.001), gestational age (R^2^ = 0.007, p = 0.01), and delivery mode (R^2^ = 0.005, p = 0.02). We next used MaAsLin2 to fit linear mixed effects models evaluating associations between these same factors and the relative abundances of specific species within the gut microbiome [20]. Substantial shifts in the composition of the gut microbiota were seen among infants during the first months of life (Fig. 1c). Specifically, we found that increasing postnatal age was associated with increases in the relative abundances of several common pathogens (*E. faecalis*: p < 0.0001; *E. coli:* p < 0.0001; *K. pneumoniae:* p < 0.0001; *K. aerogenes:* p < 0.0001) and a decrease in the relative abundance of *Staphylococcus epidermidis* (p = 0.001; Additional file 1: Table S1). Antibiotic exposures were also associated with substantial shifts in the relative abundances of several microbial species. Penicillins and aminoglycosides were associated with decreases in the relative abundances of *Bifidobacterium* and *Enterobacter* species, respectively, while cephalosporins and carbapenems were associated with losses of several *Enterobacter* and *Klebsiella* species (Fig. 2b). Interestingly, receipt of carbapenems or metronidazole was associated with increases in the relative abundances of several staphylococcal species, including *S. aureus* (carbapenems: p = 0.01), *S. capitis* (carbapenems: p < 0.0001; metronidazole: p = 0.001), and *S. epidermidis* (carbapenems: p = 0.02). Most antibiotic exposures were associated with a decline in the relative abundance of the probiotic species, *B. breve* (penicillins: p = 0.001; cephalosporins: p = 0.001; vancomycin: p = 0.02; and metronidazole: p = 0.02), while probiotic exposure was associated with enrichment of the microbiome by this species (p < 0.0001).

### Strain-level community genomic analyses identify BSI isolates within fecal samples

**Fig. 3:**
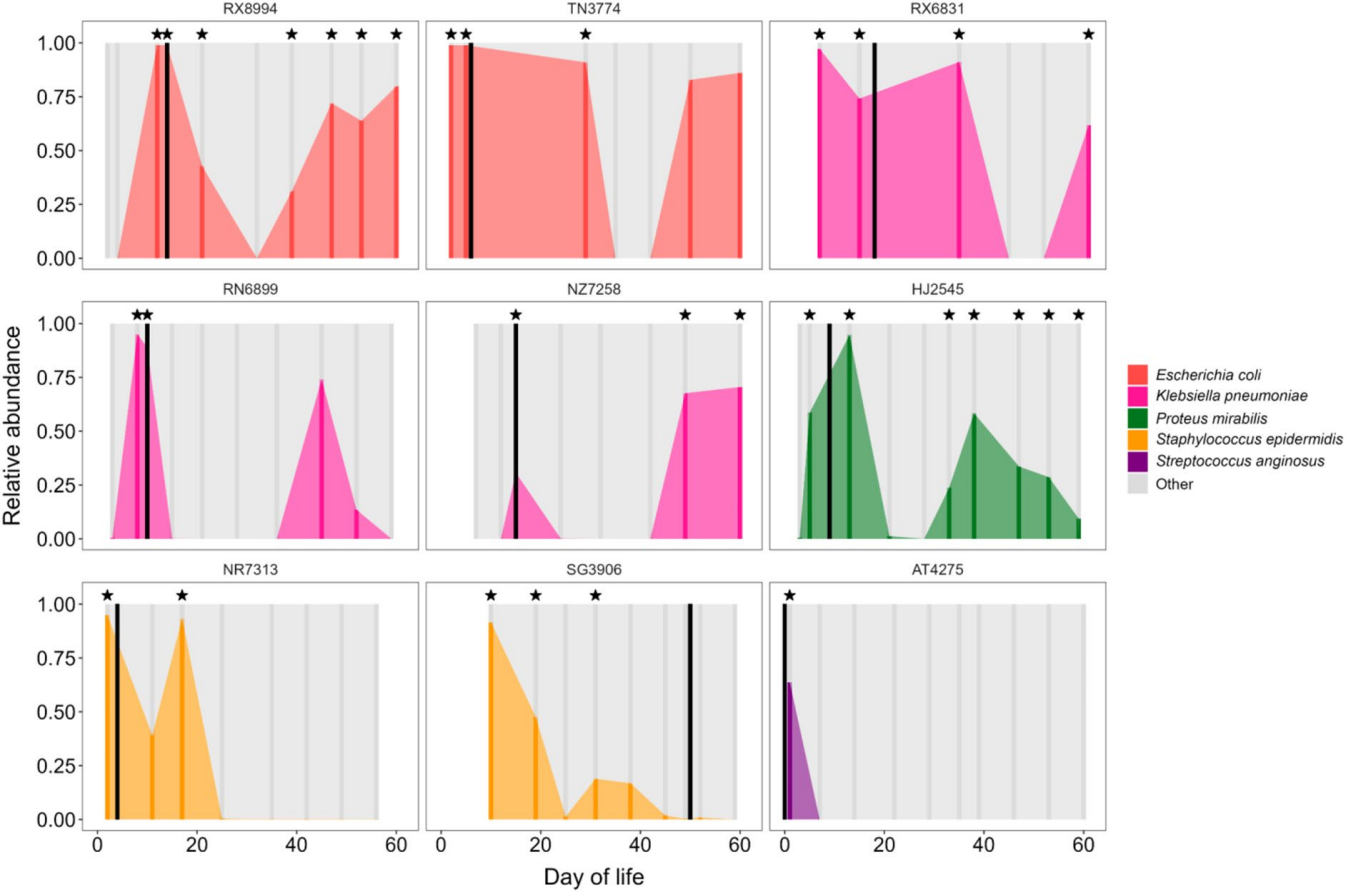
Time-series plots of gut microbiota composition among a subset of premature infants who developed BSI. The relative abundance of the BSI causative species is indicated by colored vertical bar for each fecal sample with sequencing data. Black vertical lines denote the timing of BSI episodes. Stars correspond to fecal samples in which the BSI strain was identified in strain-level analyses. Subject identifiers are atop each plot

During the study period, 16 (21%) infants developed a BSI caused by the following organisms: *Streptococcus agalactiae* (n = 5), *K. pneumoniae* (n = 3), *E. coli* (n = 2), *S. aureus* (n = 2), *S. epidermidis* (n = 2), *Proteus mirabilis* (n = 1), and *Streptococcus anginosus* (n = 1). Consistent with prior studies, we detected the BSI species at a high relative abundance in fecal samples collected prior to or following onset of the BSI episode in most infants [11, 14]. We then used inStrain to evaluate for the presence of the BSI strain in fecal samples collected from that infant. Briefly, inStrain is a program that uses metagenomic sequencing data to profile microbial populations and to perform population comparisons that account for within-population genetic heterogeneity [21]. In comparing microbial populations, inStrain calculates a measure referred to as the population average nucleotide identity (popANI), in which nucleotide substitutions are only called if the two samples being compared do not share any alleles [22]. Using inStrain and a threshold for classifying strains as being identical set at a popANI at or above 99.999%, we identified strains identical to the blood culture isolate in 12 of 16 (75%) infants who experienced a BSI (Table 3). Fecal samples containing the BSI strain were identified preceding infection onset, during antibiotic treatment for the infection and, in some infants, for a prolonged period following completion of antibiotics (Fig. 3). Notably, identical strains were identified in the gut metagenomes of all infants who developed BSI caused by typical enteric bacteria (e.g., *E. coli*, *K. pneumoniae*, *P. mirabilis*) and both infants who developed BSI caused by *S. epidermidis*, a frequent member of the gut microbiota of infants, particularly those who are breastfeeding [23]. In contrast, neither infant with a BSI caused by *S. aureus* had an identical strain identified in one or more fecal samples. Finally, the BSI strain was identified in fecal samples from 3 of 5 infants who developed BSI caused by *S. agalactiae* (group B streptococcus), a common colonizer of the infant gut, oral cavity, and other mucosal surfaces [24]. Taken together, these results support the conclusion that the gut is an important source of BSIs caused by classical enteric bacteria and other bacteria previously demonstrated to be prevalent in the infant gut.

**Table 3:** Genomic concordance of bacterial strains in fecal samples to blood culture isolates

Patient	BSI isolate	DOL of BSI	DOL of fecal sample	Coverage overlap (%)	popANI (%)	Population SNPs	conANI (%)
AD8134	*Streptococcus agalactiae*	0	No strains with popANI > 99.999% in sequenced fecal samples				
AT4275	*Streptococcus anginosus*	0	1	99.9964	100.0000	0	99.9999
HJ2545	*Proteus mirabilis*	9	5	99.9894	100.0000	0	99.9995
		9	13	99.9883	100.0000	0	99.9995
		9	33	99.8263	99.9999	4	99.9966
		9	38	99.9911	100.0000	0	99.9995
		9	47	89.0305	100.0000	0	99.9956
		9	53	94.5055	99.9998	7	99.9662
		9	59	99.8561	99.9999	4	99.9050
JU9212	*Staphylococcus aureus*	46	No strains with popANI > 99.999% in sequenced fecal samples				
NR7313	*Staphylococcus epidermidis*	4	2	99.9965	100.0000	0	99.9995
		4	17	90.3569	99.9998	4	99.9933
NZ7258	*Klebsiella pneumoniae*	15	15	99.9984	100.0000	1	99.9972
		15	49	99.9985	100.0000	0	99.9999
		15	60	99.9986	100.0000	0	99.9999
PL2596	*S. agalactiae*	65	56	99.9872	100.0000	0	99.9086
		65	61	99.9688	100.0000	0	99.9044
		65	66	99.9788	100.0000	0	99.8543
QA9843	*S. agalactiae*	32	28	99.9641	100.0000	0	99.9904
RN6899	*K. pneumoniae*	10	8	99.9991	100.0000	2	99.9998
		10	10	99.9997	100.0000	2	99.9998
RX6831	*K. pneumoniae* BC120-140220.sorted.bam BC120-140220.sorted.bam BC120-140220.sorted.bam	18	7	99.9946	100.0000	0	99.9998
		18	15	99.9944	100.0000	0	99.9998
		18	35	99.9948	100.0000	1	99.9997
		18	61	99.9943	100.0000	0	99.9995
RX8994	*Escherichia coli*	14	12	99.9909	100.0000	0	100.000
		14	14	99.9892	100.0000	0	100.000
		14	21	99.9923	100.0000	0	100.000
		14	39	99.9907	100.0000	0	100.000
		14	47	99.9895	100.0000	0	100.000
		14	53	99.9896	100.0000	0	100.000
		14	60	99.9906	100.0000	0	100.000
SG3906	*S. epidermidis*	50	10	95.0561	99.9999	2	99.9993
		50	19	99.9931	100.0000	1	99.9996
		50	31	99.9935	100.0000	0	99.9980
TN3774	*E. coli*	6	2	99.9826	100.0000	1	99.9980
		6	5	99.9565	100.0000	0	99.9973
		6	29	99.9325	100.0000	0	99.9978
YC2596	*S. aureus*	25	No strains with popANI > 99.999% in sequenced fecal samples				
YH3562	*S. agalactiae*	0	3	99.9933	100.0000	0	100.0000
		0	21	99.9983	100.0000	0	99.9975
		0	28	99.9991	100.0000	0	99.9943
		0	35	99.9959	100.0000	0	99.9905
		0	56	99.9662	100.0000	0	99.9836
ZR1676	*S. agalactiae*	35	No strains with popANI > 99.999% in sequenced fecal samples				

*BSI* bloodstream infection, *DOL* day of life, *popANI* population average nucleotide identify, *SNP* single nucleotide polymorphism, *conANI* consensus average nucleotide identity

### Strain-level comparisons of group B streptococcal BSI isolates

**Fig. 4:**
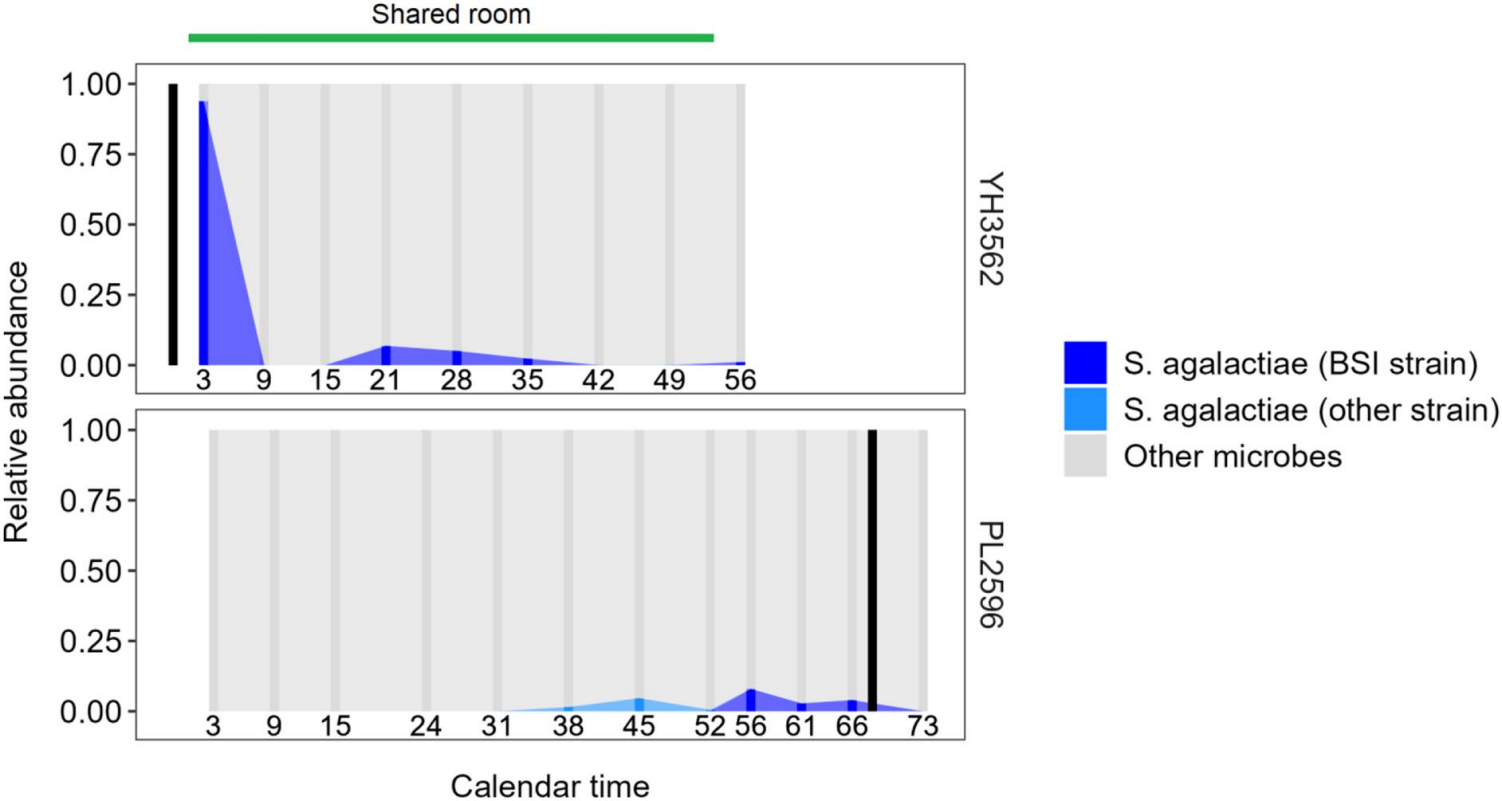
Putative transmission of a *Streptococcus agalactiae* strain between infants with an epidemiological link. Times-series plots for two infants who developed BSI from the same strain of *S. agalactiae*. The green horizontal line indicates the time period during which the infants resided in the same room and had shared healthcare providers. The relative abundance of the shared BSI strain is indicated by a dark blue vertical line for each fecal sample sequenced, while the light blue vertical lines represent the relative abundance of other *S. agalactiae* strains. Numbers below these lines correspond to the infant age in days. Black vertical lines indicate the day of the BSI episode

Despite the study period spanning more than two years, all five BSIs caused by *S. agalactiae* were identified over a two-month period in 2021. We thus hypothesized that these infections resulted from infant-to-infant transmission or acquisition from a shared environmental source within the NICU environment. To evaluate this hypothesis, we again used inStrain to compare the genomic similarity of these *S. agalactiae* strains [21]. These analyses demonstrated that the blood culture isolates from infants PL2596 and YH3562 were identical, with 100% popANI and 0 population single nucleotide polymorphisms (SNPs) with more than 99.99% genome coverage overlap (Table 4). The remaining strains were found to be distinct from this shared strain and from each other, with the strain isolated from infant ZR1676 differing from the strain shared by PL2596 and YH3562 but by only 59–60 population SNPs. To further investigate this possible strain transmission between infants PL2596 and YH3562, we obtained additional clinical and epidemiological data from these infants. Infant YH3562 presented with EOS and had *S. agalactiae* identified in a blood culture obtained on the day of birth, with gut colonization identified through at least 56 days of age. Infant PL2596 was first identified to be colonized by this *S. agalactiae* strain at 56 days of age and subsequently developed LOS on day of life 65. Notably, these infants resided in the same room within the NICU and had shared clinical providers in the week preceding BSI onset in infant PL2596 and at a time at which infant YH3562 was known to have persistent gut colonization (Fig. 4). However, we did not collect environmental samples from surfaces and equipment within the NICU to confirm the source of transmission. Finally, we compared results from investigation of these *S. agalactiae* BSIs through use of whole-genome sequencing to findings obtained from multilocus sequence typing (MLST), which remains a standard approach for evaluation of healthcare-associated infection clusters [25]. MLST analysis (Additional file 1: Table S2) classified these five strains as being from only two sequence types, ST17 and ST24, demonstrating the superior resolution of whole-genome sequencing with current genomic analysis tools relative to conventional genotyping methods.

**Table 4:** Genomic comparisons of *Streptococcus agalactiae* BSI isolates

Patient 1	Patient 2	Coverage overlap (%)	popANI (%)	Population SNPs	conANI (%)	Interpretation
AD8134	PL2596	92.6139	99.3448	12,620	99.3408	Different strains
AD8134	QA9843	95.9577	99.9896	192	99.9865	Different strains
AD8134	YH3562	92.6158	99.3446	12,623	99.3410	Different strains
AD8134	ZR1676	92.5850	99.3444	12,629	99.3406	Different strains
PL2596	QA9843	89.5652	99.3401	12,197	99.3363	Different strains
PL2596	YH3562	99.9937	100.0000	0	99.9998	Same strain
PL2596	ZR1676	99.9390	99.9971	60	99.9967	Different strains
QA9843	YH3562	89.5625	99.3399	12,201	99.3364	Different strains
QA9843	ZR1676	89.5369	99.3396	12,209	99.3359	Different strains
YH3562	ZR1676	99.9343	99.9972	59	99.9968	Different strains

*BSI* bloodstream infection, *popANI*population average nucleotide identity, *SNP* single nucleotide polymorphism, *conANI* consensus average nucleotide identity

## Discussion

In this study, we profile the gut microbiomes of a large cohort of premature infants receiving care in a Singaporean NICU. We describe the substantial impact of clinical factors, including antibiotic and probiotic exposures, on development of the premature infant gut microbiota in this setting. We additionally use strain-resolved metagenomic analyses to confirm that most infants who develop BSI harbor strains identical to the BSI culture isolate within their gut microbiota. Finally, we investigate a potential cluster of BSIs caused by *S. agalactiae* using this same analysis approach and compare these findings to those obtained using conventional genotyping methods.

Due to their medical complexity and high risk of infection, premature infants often receive multiple courses of broad-spectrum antibiotics during the first several months of life. Such antibiotic exposures during early infancy were previously demonstrated to result in prolonged disruptions of the gut microbiota [18, 19]. In particular, ampicillin, cefepime, meropenem, and vancomycin are known to substantially reduce gut microbial diversity and delay maturation of the gut microbiota [18]. In the current study, we evaluated the effects of individual antibiotics on the relative abundances of specific microbial species within the premature infant gut. We found that cephalosporin and carbapenem exposures were associated with losses of Enterobacteriaceae, and carbapenem exposure was associated with higher relative abundance of *S. epidermidis*. These findings are congruent with analyses conducted by Gibson et al. in which cefotaxime and meropenem were associated with similar changes in the abundances of Enterobacteriaceae and *S. epidermidis* within the gut microbiomes of premature infants immediately following antibiotic exposure [19]. In contrast, Fouhy et al. observed higher relative abundances of Enterobacteriaceae among full-term infants treated with ampicillin and gentamicin [26]. The inconsistent associations between antibiotic exposures and losses or gains of individual taxa in the gut microbiota were noted in a recent systematic review [27]. These discrepancies are likely due to nuances in the individual antibiotic spectra of activity and further highlight the importance of evaluating the effects of individual antibiotics on the gut microbiome. Our findings and those of related studies help inform antimicrobial stewardship efforts and the judicious use of antibiotics in premature infants.

Probiotics are frequently administered to premature infants because of data suggesting that these products may reduce the risk of necrotizing enterocolitis and LOS [28–31]. The strain used in our cohort, *B. breve* M-16 V, is among the most commonly used probiotics in infants [32]. This bacterial strain was previously demonstrated to effectively colonize the gut microbiomes of very low birth weight infants [33]. In a clinical trial conducted in the same NICU as the present study, administration of a synbiotic containing *B. breve* M-16 V, short-chain galacto-oligosaccharides, and long-chain fructo-oligosaccharides to premature infants born via Caesarian section prevented the delay in enrichment of the gut microbiota by *Bifidobacterium* typically seen among infants born by this delivery mode [34–36]. Additionally, compared to infants randomized to placebo or *B. breve* M-16 V alone, infants receiving this synbiotic had fecal pH and acetate concentrations that were more similar to those of vaginally-delivered infants [34]. Receipt of *B. breve* M-16 V almost certainly contributed to the high relative abundance of *B. breve* in the gut microbiomes of infants in our cohort; however, this species has been shown to be prevalent in the gut microbiota of other infant populations [17, 22], and we did not perform specific analyses to quantify the relative abundance of the probiotic strain in this study. Several prior studies suggested that probiotics may reduce the risk of LOS, although a recent meta-analysis reported that neither single-strain nor multistrain probiotics were associated with a reduced risk of sepsis events [31, 37–39]. Notably, all infants enrolled in the current study received the *B. breve* M-16 V probiotic, which precluded evaluation of the impact of this probiotic on the risk of BSI.

To date, most studies of the premature infant gut microbiota were conducted in the United States and Europe [14, 19, 40, 41]. There have been very few reports from Asian NICUs, and these studies have generally had small sample sizes and utilized earlier high-throughput sequencing techniques [42–45]. Despite the geographic location, the gut microbiome composition of premature infants in our study was similar to that observed in prior studies of premature infants conducted in Western countries [17, 40, 46]. While the high relative abundance of *B. breve* in our cohort was likely affected by probiotic strain administration, studies from Asia and other parts of the world previously demonstrated high relative abundances of *Bifidobacterium* early in life [17, 44–46]. In one study, *Bifidobacterium* represented nearly 25% of the newborn gut microbiota and, by 4 months of age, this had risen to a relative abundance of approximately 60% [17]. The predominance of bifidobacterial species in these studies of early infancy is likely related to breastfeeding practices given that breastfed infants have previously been shown to have higher abundances of *Bifidobacterium* compared to formula-fed infants [47]. Similar to prior studies, we also found both progressive enrichment of the infant gut microbiota by Enterobacteriaceae with increasing postnatal age and associations between microbial composition and gestational age, delivery mode, and diet [19, 40, 43, 45]. Interestingly, we also found that the gut microbiome composition of premature infants in this study differed according to race, an association that could be related in part to differences in maternal diet and cultural practices known to affect the microbiome [48, 49]. In summary, our study provides insight into the gut microbiome of a population underrepresented in the literature and confirms that assembly of the premature infant gut microbiome in this setting largely parallels what has been reported in other parts of the world.

We used a recently developed metagenomic data analysis tool, inStrain, to perform comparisons of bacterial strains in mixed gut communities and BSI culture isolates, confirming that the premature infant gut serves as a reservoir for organisms that cause BSI [7, 21, 41]. Similar analyses were recently conducted in other high-risk populations, including pediatric and adult hematopoietic stem cell transplant recipients and children with short bowel syndrome [50–52]. Notably, while we detected the BSI causative strain in the gut microbiota of several infants who developed EOS, this is unlikely to be the primary source of infection and more likely to represent an additional site of colonization following inoculation during the birthing process [22]. We also described the temporal patterns of gut colonization by BSI strains, demonstrating that the presence of these strains in the premature infant gut typically precedes onset of BSI episodes and frequently extends far past completion of antibiotic therapy. This observation leads to several clinical questions: (1) could the premature infant gut microbiota be used to identify premature infants who are at high risk of developing a BSI?; (2) are there interventions that could be implemented at the time of pathogen colonization or at a threshold abundance that could prevent BSI in this patient population?; (3) to what extent does maturation of the gut microbiota prevent recurrent BSI episodes among infants with persistent colonization by BSI organisms following antibiotic treatment? As generating metagenomic data becomes less expensive and analyses become increasingly high-throughput, these tools will inevitably be used to answer these questions and to inform the care of premature infants and other patient populations.

Our application of whole-genome sequencing to a cluster of *S. agalactiae* BSIs illustrates the limitations of current genotyping methods for infection prevention and control. In particular, we compared genome sequencing to MLST, the latter of which has been the standard tool for investigation of infection clusters for decades [25]. While genome sequencing enables analysis of entire genomes, MLST typically involves sequencing of seven housekeeping genes that represent less than 1% of bacterial genomes [53]. The result is that whole-genome sequencing has far higher resolution for strain comparisons than MLST and other conventional genotyping methods, as has been demonstrated by several prior studies [54–56]. For instance, whole-genome sequencing recently demonstrated that *K. pneumoniae* sequence type 258, a frequent cause of healthcare-associated carbapenem-resistant Enterobacteriaceae infection, represents two distinct clades, disproving the hypothesis that the widespread infections attributed to this organism are caused by a single bacterial clone [56]. Although limited to investigation of a single infection cluster, our analysis similarly demonstrated the superiority of whole-genome sequencing for the differentiation of *S. agalactiae* strains. In a healthcare system increasingly strained for resources, use of whole-genome sequencing has the potential to inform infection control policies and improve allocation of the limited resources available for investigation of and response to infection clusters.

Our study has several limitations. Although we performed metagenomic sequencing on weekly samples collected from 75 premature infants, analyses may have been limited by the frequency and depth of sequencing of fecal samples. For instance, we may have underestimated the rapidity at which the gut microbiota changes during the first months of life, and higher sequencing depth may have enabled detection of additional BSI strains present within the gut microbiota at low abundance. Analyses evaluating for gut colonization among infants who experienced a BSI were limited by the relatively small number of infections that occurred in this cohort. Similarly, our comparison of whole-genome sequencing to MLST was limited to a single infection cluster. Finally, while we used a recently developed tool that represents a significant advance for comparison of microbial populations, our analyses did not quantify the relative abundances of specific strains within these populations.

## Conclusions

To conclude, this study conducted in a Singaporean NICU provides data on the premature infant gut microbiota in an understudied geographical area and demonstrates the roles of antibiotics and probiotics in shaping gut microbiome maturation among premature infants in this setting. Additionally, we provide conclusive genomic data that support the gut as a reservoir for BSI among premature infants. Finally, this study adds to a growing body of literature demonstrating the potential utility of whole-genome sequencing for hospital infection prevention and control. These data provide a basis for future studies evaluating the utility of metagenomic sequencing for the identification of premature infants at high risk of BSI.

## Methods

### Study design and setting

This was a prospective cohort study of infants born at or before 30 weeks gestational age and admitted to the KKH NICU between June 2019 and May 2021. KKH is an 830-bed specialized maternal and pediatric hospital in Singapore that houses a 40-bed level 4 NICU. Infants were enrolled within 72 h of birth and followed through 65 days of age, hospital discharge, or death. First-line empiric antibiotics for EOS and LOS in the KKH NICU are penicillin plus gentamicin and cloxacillin plus amikacin, respectively. All infants routinely receive *B. breve* strain M-16 V (Morinaga Milk Industry Co., Tokyo, Japan) from the time they start enteral feeds through 36 weeks adjusted gestational age. Legal guardians provided written informed consent for infants to participate in the study. The study protocol was approved by the Singhealth Institutional Review Board (CIRB Ref. No. 2017/2117).

### Study procedures

Demographic and clinical data were collected from the KKH VLBW registry and infant medical records. BSI was defined as a clinical episode with one or more positive blood cultures in the presence of signs or symptoms of infection. Blood cultures with coagulase-negative staphylococci, *Bacillus*, *Corynebacterium*, *Micrococcus*, and *Propionibacterium* species were considered contaminants unless two or more cultures grew the organism or the infant showed signs of sepsis and received intravenous antibiotics for at least five days. Fecal samples were collected into eNAT® (Copan Italia, Brescia, Italy), a guanidine thiocyanate-based medium that inactivates microorganisms and stabilizes microbial DNA [57]. Culture isolates from BSIs occurring among enrolled infants were obtained from the hospital microbiology laboratory and stored using Microbank™ cryopreservation vials (Pro-Lab Diagnostics, Richmond Hill, Ontario, Canada). Fecal samples and blood culture isolates were stored at −80 °C prior to being shipped on dry ice to Duke University. The Duke Microbiome Core extracted DNA from fecal samples and blood culture isolates using PowerSoil Pro Kits (Qiagen, Germantown, MD). The Duke Sequencing and Genomic Technologies Shared Resource then constructed libraries using Illumina DNA Library Prep Kits (Illumina, San Diego, CA) and sequenced these libraries on a NovaSeq6000 instrument (Illumina) as 150-bp paired-end reads. Samples were sequenced in two batches with inclusion of negative extraction and library preparation controls and the ZymoBIOMICS Gut Microbiome Standard (Zymo Research Corp, Irvine, CA) as a positive extraction control. One negative extraction control contained no quality-filtered reads, while the remaining negative controls contained 4–177 microbial reads, with the most abundant genera being *Micrococcus* and *Streptococcus*.

### Bioinformatic processing of sequencing reads from fecal samples and blood culture isolates

Raw sequencing reads from fecal samples and blood culture isolates were quality-filtered and removed of host contamination (hg19 human reference genome) using KneadData v0.10.0. Kraken2 v2.1.2 was used for taxonomic classification and was run in paired-end mode with default parameters against a database of all complete bacterial, fungal, archaeal, and viral genomes in the NCBI Reference Sequence Database as of February 2023 [58–60]. The abundances of species were estimated using Bracken v2.6.2 with database construction performed using a read length of 150 bp and the default k-mer length of 35 bp. [61] Quality-filtered sequencing reads from blood culture isolates were assembled using SPAdes v3.15.3 [62]. Contigs shorter than 1000 bp were removed from these genome assemblies using BBMap v38.93 [63]. All genomes were determined to have ≥ 99% completeness and < 1% contamination as determined by checkM v1.1.3, with a median (IQR) genome coverage of 774 (356–1128) as calculated by BBMap (Additional file 1: Table S3) [64]. We pruned fecal samples with fewer than 10,000 quality-filtered sequencing reads.

### Statistical analyses

We estimated alpha diversity using the *phyloseq* R package v1.42.0 and used mixed effects regression to evaluate associations between week of life and the Shannon diversity index (linear regression) and number of observed microbial species (negative binomial regression) in fecal samples [65]. Using the adonis2 function of the *vegan* package v2.6–4, we performed PERMANOVA on Bray–Curtis distances to evaluate associations between clinical factors and overall gut microbiome composition [66]. Analyses considered postnatal age, gestational age at birth, birthweight, patient race, sex, delivery mode, diet (breast milk, formula, or both), postnatal antibiotic exposures, and probiotic exposure with participant as a blocking factor to account for repeated sampling. Additional PERMANOVAs were performed on these clinical factors stratified by postnatal age. We then used the *Maaslin2* package v1.12.0 to fit a linear mixed effects model evaluating associations between clinical factors, including specific antibiotic exposures, and the relative abundances of specific microbial species [20]. This model included postnatal age, gestational age at birth, birthweight, delivery mode, patient race, sex, percent of enteral feeds (expressed breast milk, pasteurized donor human milk, and formula), postnatal antibiotic exposures, and probiotic exposure. We included subject as a random effect and limited analyses to species that had a minimum mean relative abundance of 0.01% and a sample prevalence of at least 10%. Infants were considered exposed to an antibiotic or the probiotic if they received at least one dose since collection of the last sequenced fecal sample. If a subject started antibiotics or the probiotic on the day of fecal sample collection, the exposure was assigned to the next sequenced sample. Antibiotic exposures included antibiotics that were administered to at least 5% of the study population: aminoglycosides, carbapenems, cephalosporins, metronidazole, penicillins, and vancomycin.

### Comparison of bacterial strains in blood culture isolates and fecal samples

We used inStrain v1.6.4 to perform genomic comparisons of bacterial strains in fecal samples and blood culture isolates [22]. To evaluate for the presence of the BSI strain in fecal samples from the same infant, we first mapped fecal and blood culture metagenomic sequencing reads against the assembled blood culture isolate genome using BWA v0.7.17 [67]. Pairs of samples with at least 50% coverage breadth at a depth of five or more reads were compared to identify SNPs and to calculate popANI values. To compare the genomic similarity of blood culture isolates from different infants, we de-replicated genomes using dRep v3.4.1 with the primary clustering threshold set to an average nucleotide identity of 95% [68]. We then mapped metagenomic sequencing reads from blood culture isolates against this de-replicated set of genomes using BWA and used inStrain to identify SNPs and calculate popANI values for each pairwise comparison [21, 67]. For all analyses, we set the threshold for classifying strains as being identical at a popANI of 99.999%, which was previously proposed as a highly stringent threshold for strain sameness within the limit of detection for metagenomic analyses [22].

### Genotypic analyses using multilocus sequence typing

DNA from *S. agalactiae* blood culture isolates was extracted using the ZymoBIOMICS DNA Mini Prep kit (Zymo Research Corp). Extracted DNA for each BSI isolate was then used as template DNA in PCR assays targeting housekeeping genes (adhP, pheS, atr, glnA, sdhA, glcK, tkt), as described previously [69]. PCR products underwent Sanger sequencing and the resulting chromatograms were submitted to the public databases for molecular typing and microbial genome diversity (PubMLST) for allele determination and identification of strain sequence type [70].

### Supplementary Information

**Additional file 1: Table S1**. Significant associations of clinical factors and antibiotic exposures and the relative abundances of microbial species within the gut microbiome. **Table S2.** Multilocus sequence typing of *Streptococcus agalactiae* BSI isolates. **Table S3. **Statistics for genomes generated from BSI isolates.

## Data Availability

The metagenomic data analyzed in this study are available on the NCBI Sequence Read Archive (BioProject PRJNA947616). The de-identified metadata and script used for data analyses are available upon request.
