# Supplementary material for: Strain-resolved metagenomic analysis of the gut as a reservoir for bloodstream infection pathogens among premature infants in Singapore
Source: Gut Pathog. 2023 Nov 16;15:55. doi: 10.1186/s13099-023-00583-8 (PMC10652614; doi:10.1186/s13099-023-00583-8)
Supplement: Supplementary file 1 — Additional file 1: Table S1. Significant associations of clinical factors and antibiotic exposures and the relative abundances of microbial species within the gut microbiome. Table S2. Multilocus sequence typing of Streptococcus agalactiae BSI isolates. Table S3. Statistics for genomes generated from BSI isolates. [file 13099_2023_583_MOESM1_ESM.docx]

| **Table S1**. Significant associations of clinical factors and antibiotic exposures and the relative abundances of microbial species within the gut microbiome | | | | | | |
| --- | --- | --- | --- | --- | --- | --- |
| **Covariate** | | **Species** | **Effect** | **Standard Error** | **P Value** | **Q Value** |
| **Antibiotic Exposure** | |  |  |  |  |  |
|  | Aminoglycoside | *Enterobacter asburiae* | -1.42334 | 0.49376 | 0.00411 | 0.041 |
|  | Aminoglycoside | *Enterobacter bugandensis* | -1.01688 | 0.40717 | 0.01282 | 0.09907 |
|  | Aminoglycoside | *Enterobacter cancerogenus* | -0.99698 | 0.38046 | 0.00903 | 0.07461 |
|  | Aminoglycoside | *Enterobacter hormaechei* | -1.90775 | 0.73078 | 0.00929 | 0.07651 |
|  | Aminoglycoside | *Enterobacter kobei* | -1.73376 | 0.55988 | 0.00206 | 0.02144 |
|  | Carbapenem | *Citrobacter freundii* | -1.51153 | 0.45507 | 0.00096 | 0.01073 |
|  | Carbapenem | *Citrobacter koseri* | -1.5507 | 0.41431 | 2.00E-04 | 0.00276 |
|  | Carbapenem | *Enterobacter asburiae* | -1.06076 | 0.39373 | 0.00729 | 0.06482 |
|  | Carbapenem | *Enterobacter cloacae* | -1.58704 | 0.49754 | 0.00151 | 0.01639 |
|  | Carbapenem | *Enterobacter hormaechei* | -1.59456 | 0.58258 | 0.00641 | 0.05959 |
|  | Carbapenem | *Enterobacter roggenkampii* | -1.34043 | 0.5137 | 0.00933 | 0.07657 |
|  | Carbapenem | *Enterococcus faecalis* | -2.71736 | 0.77077 | 0.00046 | 0.00554 |
|  | Carbapenem | *Escherichia coli* | -4.38574 | 0.77034 | 0 | 0 |
|  | Carbapenem | *Klebsiella aerogenes* | -2.44191 | 0.54881 | 1.00E-05 | 2.00E-04 |
|  | Carbapenem | *Klebsiella africana* | -1.93645 | 0.39019 | 0 | 2.00E-05 |
|  | Carbapenem | *Klebsiella grimontii* | -1.63943 | 0.39195 | 3.00E-05 | 0.00058 |
|  | Carbapenem | *Klebsiella michiganensis* | -2.48145 | 0.48519 | 0 | 1.00E-05 |
|  | Carbapenem | *Klebsiella oxytoca* | -2.55878 | 0.49679 | 0 | 1.00E-05 |
|  | Carbapenem | *Klebsiella pneumoniae* | -4.4319 | 0.85762 | 0 | 1.00E-05 |
|  | Carbapenem | *Klebsiella quasipneumoniae* | -3.41497 | 0.59104 | 0 | 0 |
|  | Carbapenem | *Klebsiella quasivariicola* | -2.2587 | 0.41156 | 0 | 0 |
|  | Carbapenem | *Klebsiella sp LY* | -1.01547 | 0.29096 | 0.00052 | 0.00623 |
|  | Carbapenem | *Klebsiella sp WP4 W18 ESBL 05* | -0.8907 | 0.29674 | 0.00281 | 0.02864 |
|  | Carbapenem | *Klebsiella variicola* | -2.62826 | 0.57941 | 1.00E-05 | 0.00013 |
|  | Carbapenem | *Raoultella ornithinolytica* | -1.43098 | 0.32829 | 2.00E-05 | 0.00029 |
|  | Carbapenem | *Salmonella enterica* | -2.53769 | 0.48314 | 0 | 0 |
|  | Carbapenem | *Shigella flexneri* | -1.87756 | 0.4984 | 0.00018 | 0.00253 |
|  | Carbapenem | *Staphylococcus aureus* | 2.07196 | 0.62853 | 0.00104 | 0.01157 |
|  | Carbapenem | *Staphylococcus capitis* | 2.64214 | 0.49568 | 0 | 0 |
|  | Carbapenem | *Staphylococcus epidermidis* | 2.44999 | 0.76358 | 0.00141 | 0.01539 |
|  | Carbapenem | *Staphylococcus haemolyticus* | 1.78098 | 0.52413 | 0.00073 | 0.00829 |
|  | Cephalosporin | *Bifidobacterium bifidum* | -0.74208 | 0.26549 | 0.00536 | 0.05135 |
|  | Cephalosporin | *Bifidobacterium breve* | -2.36082 | 0.57192 | 4.00E-05 | 0.00072 |
|  | Cephalosporin | *Klebsiella aerogenes* | -1.38685 | 0.43797 | 0.00163 | 0.01746 |
|  | Cephalosporin | *Klebsiella africana* | -1.57942 | 0.31102 | 0 | 1.00E-05 |
|  | Cephalosporin | *Klebsiella grimontii* | -1.16957 | 0.31067 | 0.00018 | 0.00253 |
|  | Cephalosporin | *Klebsiella michiganensis* | -1.61502 | 0.38031 | 3.00E-05 | 0.00046 |
|  | Cephalosporin | *Klebsiella oxytoca* | -2.11119 | 0.39415 | 0 | 0 |
|  | Cephalosporin | *Klebsiella pneumoniae* | -3.38406 | 0.67408 | 0 | 1.00E-05 |
|  | Cephalosporin | *Klebsiella quasipneumoniae* | -2.61361 | 0.46793 | 0 | 0 |
|  | Cephalosporin | *Klebsiella quasivariicola* | -1.35156 | 0.32794 | 4.00E-05 | 0.00074 |
|  | Cephalosporin | *Klebsiella sp WP4 W18 ESBL 05* | -0.79846 | 0.23703 | 0.00081 | 0.00917 |
|  | Cephalosporin | *Klebsiella variicola* | -2.14682 | 0.45848 | 0 | 7.00E-05 |
|  | Cephalosporin | *Raoultella ornithinolytica* | -0.96859 | 0.2592 | 0.00021 | 0.00278 |
|  | Metronidazole | *Bifidobacterium breve* | -3.22602 | 1.01293 | 0.00153 | 0.01655 |
|  | Metronidazole | *Staphylococcus capitis* | 2.70042 | 0.68493 | 9.00E-05 | 0.0014 |
|  | Penicillins | *Bifidobacterium breve* | -3.74906 | 0.91744 | 5.00E-05 | 0.00083 |
|  | Penicillins | *Bifidobacterium longum* | -2.25361 | 0.73492 | 0.00227 | 0.02353 |
|  | Penicillins | *Enterococcus faecalis* | -2.45243 | 0.9579 | 0.01074 | 0.0849 |
|  | Vancomycin | *Bifidobacterium breve* | -4.57862 | 1.45034 | 0.00168 | 0.01796 |
|  | Vancomycin | *Clostridium perfringens* | -3.63812 | 1.37384 | 0.00834 | 0.06983 |
|  | Vancomycin | *Enterococcus faecalis* | -4.25307 | 1.502 | 0.00481 | 0.04671 |
|  | Vancomycin | *Enterococcus faecium* | -3.39076 | 0.92548 | 0.00027 | 0.00355 |
| **Clinical Factors** | |  |  |  |  |  |
|  | Birthweight | *Bacillus cereus* | 0.61799 | 0.2352 | 0.01054 | 0.0839 |
|  | Birthweight | *Bacillus mycoides* | 0.52835 | 0.20097 | 0.01051 | 0.0839 |
|  | Birthweight | *Caloramator sp E03* | 0.60112 | 0.22656 | 0.00985 | 0.07972 |
|  | Birthweight | *Cellulosilyticum sp WCF 2* | 0.62328 | 0.22622 | 0.00747 | 0.06547 |
|  | Birthweight | *Clostridium acetobutylicum* | 0.76229 | 0.27307 | 0.00676 | 0.06154 |
|  | Birthweight | *Clostridium argentinense* | 0.80976 | 0.29722 | 0.00812 | 0.06868 |
|  | Birthweight | *Clostridium autoethanogenum* | 0.65753 | 0.20771 | 0.00231 | 0.02378 |
|  | Birthweight | *Clostridium baratii* | 1.15766 | 0.41646 | 0.00698 | 0.06307 |
|  | Birthweight | *Clostridium beijerinckii* | 1.19316 | 0.45601 | 0.01087 | 0.08571 |
|  | Birthweight | *Clostridium bornimense* | 0.93942 | 0.34937 | 0.00896 | 0.07423 |
|  | Birthweight | *Clostridium cadaveris* | 0.89159 | 0.33647 | 0.00995 | 0.0803 |
|  | Birthweight | *Clostridium carboxidivorans* | 0.67306 | 0.25451 | 0.01009 | 0.08086 |
|  | Birthweight | *Clostridium cellulovorans* | 0.78266 | 0.28326 | 0.00731 | 0.06482 |
|  | Birthweight | *Clostridium chauvoei* | 0.98052 | 0.36044 | 0.00823 | 0.06918 |
|  | Birthweight | *Clostridium cochlearium* | 0.68332 | 0.24992 | 0.00791 | 0.06744 |
|  | Birthweight | *Clostridium diolis* | 0.92178 | 0.32815 | 0.00644 | 0.05959 |
|  | Birthweight | *Clostridium drakei* | 0.61637 | 0.22566 | 0.00799 | 0.06788 |
|  | Birthweight | *Clostridium estertheticum* | 0.78585 | 0.27948 | 0.00638 | 0.05959 |
|  | Birthweight | *Clostridium formicaceticum* | 0.52417 | 0.20539 | 0.01286 | 0.09907 |
|  | Birthweight | *Clostridium gasigenes* | 0.97325 | 0.35511 | 0.00777 | 0.06673 |
|  | Birthweight | *Clostridium intestinale* | 0.93483 | 0.3365 | 0.00701 | 0.06313 |
|  | Birthweight | *Clostridium isatidis* | 0.95553 | 0.34893 | 0.00783 | 0.06696 |
|  | Birthweight | *Clostridium kluyveri* | 0.77908 | 0.28414 | 0.00775 | 0.06673 |
|  | Birthweight | *Clostridium novyi* | 0.82075 | 0.29228 | 0.00645 | 0.05959 |
|  | Birthweight | *Clostridium pasteurianum* | 0.84104 | 0.30533 | 0.00748 | 0.06547 |
|  | Birthweight | *Clostridium saccharobutylicum* | 1.16935 | 0.44028 | 0.00979 | 0.07951 |
|  | Birthweight | *Clostridium saccharoperbutylacetonicum* | 1.16532 | 0.43137 | 0.00866 | 0.07206 |
|  | Birthweight | *Clostridium scatologenes* | 0.66918 | 0.22977 | 0.00482 | 0.04671 |
|  | Birthweight | *Clostridium septicum* | 1.02034 | 0.37017 | 0.00745 | 0.06547 |
|  | Birthweight | *Clostridium sp AWRP* | 0.60566 | 0.23483 | 0.01201 | 0.09402 |
|  | Birthweight | *Clostridium sp C5S11* | 1.10433 | 0.43006 | 0.01237 | 0.09597 |
|  | Birthweight | *Clostridium sp CT4* | 0.92012 | 0.33221 | 0.00718 | 0.06415 |
|  | Birthweight | *Clostridium sp DL VIII* | 1.08395 | 0.41316 | 0.01069 | 0.08478 |
|  | Birthweight | *Clostridium sp JN 1* | 0.65987 | 0.24229 | 0.00814 | 0.06868 |
|  | Birthweight | *Clostridium sp JN 9* | 0.68315 | 0.23853 | 0.00551 | 0.05251 |
|  | Birthweight | *Clostridium sp JN500901* | 0.66077 | 0.23836 | 0.00712 | 0.06387 |
|  | Birthweight | *Clostridium sp MF28* | 1.14722 | 0.40666 | 0.00622 | 0.0586 |
|  | Birthweight | *Clostridium sp SYSU GA15002* | 0.67914 | 0.24051 | 0.00617 | 0.0584 |
|  | Birthweight | *Clostridium taeniosporum* | 1.12478 | 0.42195 | 0.00953 | 0.07768 |
|  | Birthweight | *Clostridium tetani* | 0.7382 | 0.26833 | 0.00755 | 0.06573 |
|  | Birthweight | *Clostridium tyrobutyricum* | 0.66545 | 0.24915 | 0.00939 | 0.07681 |
|  | Birthweight | *Cutibacterium acnes* | -0.8603 | 0.31765 | 0.00851 | 0.071 |
|  | Birthweight | *Fusobacterium nucleatum* | 0.59739 | 0.21701 | 0.00749 | 0.06547 |
|  | Birthweight | *Hathewaya histolytica* | 0.6768 | 0.23238 | 0.00482 | 0.04671 |
|  | Birthweight | *Paeniclostridium sordellii* | 0.80836 | 0.27718 | 0.00476 | 0.04671 |
|  | Birthweight | *Paraclostridium bifermentans* | 0.71841 | 0.24647 | 0.00477 | 0.04671 |
|  | Birthweight | *Romboutsia hominis* | 0.69088 | 0.25207 | 0.00777 | 0.06673 |
|  | Birthweight | *Romboutsia ilealis* | 0.70162 | 0.25012 | 0.00652 | 0.05998 |
|  | Birthweight | *Romboutsia sp CE17* | 0.70213 | 0.25539 | 0.00759 | 0.06586 |
|  | Birthweight | *Veillonella atypica* | 1.25409 | 0.41655 | 0.00361 | 0.03639 |
|  | Birthweight | *Veillonella dispar* | 1.10847 | 0.35219 | 0.00239 | 0.02447 |
|  | Birthweight | *Veillonella parvula* | 1.6078 | 0.54984 | 0.00467 | 0.04633 |
|  | Birthweight | *Veillonella rodentium* | 0.70352 | 0.2177 | 0.00185 | 0.01971 |
|  | Birthweight | *Veillonella sp T1 7* | 1.07488 | 0.32793 | 0.00162 | 0.01742 |
|  | Day of Life | *Amedibacterium intestinale* | 0.66149 | 0.07818 | 0 | 0 |
|  | Day of Life | *Arcobacter cryaerophilus* | 0.66168 | 0.0784 | 0 | 0 |
|  | Day of Life | *Bacillus cereus* | 0.71477 | 0.08905 | 0 | 0 |
|  | Day of Life | *Bacillus megaterium* | 0.69663 | 0.07986 | 0 | 0 |
|  | Day of Life | *Bacillus mycoides* | 0.66003 | 0.07484 | 0 | 0 |
|  | Day of Life | *Caloramator sp E03* | 0.72482 | 0.08282 | 0 | 0 |
|  | Day of Life | *Caprobacter fermentans* | 0.73026 | 0.08297 | 0 | 0 |
|  | Day of Life | *Cellulosilyticum sp WCF 2* | 0.7197 | 0.07989 | 0 | 0 |
|  | Day of Life | *Citrobacter freundii* | 0.91203 | 0.12292 | 0 | 0 |
|  | Day of Life | *Citrobacter koseri* | 0.43775 | 0.11148 | 1.00E-04 | 0.00149 |
|  | Day of Life | *Citrobacter portucalensis* | 0.4055 | 0.0902 | 1.00E-05 | 0.00016 |
|  | Day of Life | *Citrobacter sp Y3* | 0.41433 | 0.06328 | 0 | 0 |
|  | Day of Life | *Citrobacter youngae* | 0.48515 | 0.07729 | 0 | 0 |
|  | Day of Life | *Clostridioides difficile* | 1.6869 | 0.14995 | 0 | 0 |
|  | Day of Life | *Clostridium acetobutylicum* | 0.89058 | 0.09292 | 0 | 0 |
|  | Day of Life | *Clostridium argentinense* | 0.95653 | 0.10068 | 0 | 0 |
|  | Day of Life | *Clostridium autoethanogenum* | 0.67011 | 0.07293 | 0 | 0 |
|  | Day of Life | *Clostridium baratii* | 1.38885 | 0.1358 | 0 | 0 |
|  | Day of Life | *Clostridium beijerinckii* | 1.60173 | 0.15536 | 0 | 0 |
|  | Day of Life | *Clostridium bornimense* | 1.12525 | 0.11818 | 0 | 0 |
|  | Day of Life | *Clostridium botulinum* | 1.66668 | 0.15612 | 0 | 0 |
|  | Day of Life | *Clostridium butyricum* | 1.81533 | 0.18895 | 0 | 0 |
|  | Day of Life | *Clostridium cadaveris* | 1.16983 | 0.11151 | 0 | 0 |
|  | Day of Life | *Clostridium carboxidivorans* | 0.78784 | 0.08792 | 0 | 0 |
|  | Day of Life | *Clostridium cellulovorans* | 0.88047 | 0.09568 | 0 | 0 |
|  | Day of Life | *Clostridium chauvoei* | 1.21008 | 0.11687 | 0 | 0 |
|  | Day of Life | *Clostridium cochlearium* | 0.79352 | 0.08504 | 0 | 0 |
|  | Day of Life | *Clostridium diolis* | 1.01361 | 0.11293 | 0 | 0 |
|  | Day of Life | *Clostridium drakei* | 0.74593 | 0.0804 | 0 | 0 |
|  | Day of Life | *Clostridium estertheticum* | 0.88492 | 0.09615 | 0 | 0 |
|  | Day of Life | *Clostridium formicaceticum* | 0.65507 | 0.07908 | 0 | 0 |
|  | Day of Life | *Clostridium gasigenes* | 1.14849 | 0.11618 | 0 | 0 |
|  | Day of Life | *Clostridium innocuum* | 0.83382 | 0.09852 | 0 | 0 |
|  | Day of Life | *Clostridium intestinale* | 1.09868 | 0.11116 | 0 | 0 |
|  | Day of Life | *Clostridium isatidis* | 1.1761 | 0.11492 | 0 | 0 |
|  | Day of Life | *Clostridium kluyveri* | 0.94866 | 0.10188 | 0 | 0 |
|  | Day of Life | *Clostridium ljungdahlii* | 0.80866 | 0.09158 | 0 | 0 |
|  | Day of Life | *Clostridium novyi* | 0.92014 | 0.09874 | 0 | 0 |
|  | Day of Life | *Clostridium pasteurianum* | 0.98837 | 0.10402 | 0 | 0 |
|  | Day of Life | *Clostridium perfringens* | 2.38907 | 0.19051 | 0 | 0 |
|  | Day of Life | *Clostridium saccharobutylicum* | 1.36929 | 0.14855 | 0 | 0 |
|  | Day of Life | *Clostridium saccharoperbutylacetonicum* | 1.37767 | 0.14446 | 0 | 0 |
|  | Day of Life | *Clostridium scatologenes* | 0.74881 | 0.07993 | 0 | 0 |
|  | Day of Life | *Clostridium scindens* | 0.79099 | 0.0893 | 0 | 0 |
|  | Day of Life | *Clostridium septicum* | 1.23864 | 0.12033 | 0 | 0 |
|  | Day of Life | *Clostridium sp AWRP* | 0.70159 | 0.08539 | 0 | 0 |
|  | Day of Life | *Clostridium sp C5S11* | 1.33094 | 0.14477 | 0 | 0 |
|  | Day of Life | *Clostridium sp CT4* | 1.12271 | 0.11359 | 0 | 0 |
|  | Day of Life | *Clostridium sp DL VIII* | 1.35911 | 0.13991 | 0 | 0 |
|  | Day of Life | *Clostridium sp JN 1* | 0.77359 | 0.08608 | 0 | 0 |
|  | Day of Life | *Clostridium sp JN 9* | 0.77548 | 0.08407 | 0 | 0 |
|  | Day of Life | *Clostridium sp JN500901* | 0.75478 | 0.08285 | 0 | 0 |
|  | Day of Life | *Clostridium sp M62 1* | 0.6714 | 0.07961 | 0 | 0 |
|  | Day of Life | *Clostridium sp MF28* | 1.27489 | 0.13696 | 0 | 0 |
|  | Day of Life | *Clostridium sp SYSU GA15002* | 0.76655 | 0.08418 | 0 | 0 |
|  | Day of Life | *Clostridium sporogenes* | 1.49855 | 0.13895 | 0 | 0 |
|  | Day of Life | *Clostridium taeniosporum* | 1.36507 | 0.13902 | 0 | 0 |
|  | Day of Life | *Clostridium tetani* | 0.85733 | 0.09175 | 0 | 0 |
|  | Day of Life | *Clostridium tyrobutyricum* | 0.79558 | 0.08924 | 0 | 0 |
|  | Day of Life | *Cutibacterium avidum* | 0.84725 | 0.18013 | 0 | 7.00E-05 |
|  | Day of Life | *Enterobacter asburiae* | 0.33116 | 0.1061 | 0.0019 | 0.02014 |
|  | Day of Life | *Enterobacter cloacae complex sp ECNIH7* | 0.17437 | 0.06993 | 0.01296 | 0.09958 |
|  | Day of Life | *Enterobacter hormaechei* | 0.51114 | 0.15741 | 0.00124 | 0.0136 |
|  | Day of Life | *Enterobacter roggenkampii* | 0.34741 | 0.13838 | 0.01236 | 0.09597 |
|  | Day of Life | *Enterobacter sp N18 03635* | 0.29789 | 0.07119 | 3.00E-05 | 0.00058 |
|  | Day of Life | *Enterococcus avium* | 1.2562 | 0.13604 | 0 | 0 |
|  | Day of Life | *Enterococcus faecalis* | 2.25155 | 0.20833 | 0 | 0 |
|  | Day of Life | *Enterococcus faecium* | 1.24794 | 0.12837 | 0 | 0 |
|  | Day of Life | *Escherichia albertii* | 0.85699 | 0.10493 | 0 | 0 |
|  | Day of Life | *Escherichia coli* | 1.7956 | 0.20806 | 0 | 0 |
|  | Day of Life | *Escherichia fergusonii* | 0.82074 | 0.10358 | 0 | 0 |
|  | Day of Life | *Escherichia marmotae* | 0.77565 | 0.09339 | 0 | 0 |
|  | Day of Life | *Escherichia sp E4742* | 0.67041 | 0.08855 | 0 | 0 |
|  | Day of Life | *Finegoldia magna* | 0.84013 | 0.12397 | 0 | 0 |
|  | Day of Life | *Fusobacterium nucleatum* | 0.58654 | 0.08763 | 0 | 0 |
|  | Day of Life | *Hathewaya histolytica* | 0.77603 | 0.07939 | 0 | 0 |
|  | Day of Life | *Klebsiella aerogenes* | 0.86837 | 0.14802 | 0 | 0 |
|  | Day of Life | *Klebsiella africana* | 0.42446 | 0.10528 | 6.00E-05 | 0.00101 |
|  | Day of Life | *Klebsiella grimontii* | 0.34959 | 0.10598 | 0.00104 | 0.01157 |
|  | Day of Life | *Klebsiella michiganensis* | 0.65964 | 0.13182 | 0 | 2.00E-05 |
|  | Day of Life | *Klebsiella oxytoca* | 0.71779 | 0.13428 | 0 | 0 |
|  | Day of Life | *Klebsiella pneumoniae* | 1.2917 | 0.23271 | 0 | 0 |
|  | Day of Life | *Klebsiella quasipneumoniae* | 1.11197 | 0.15989 | 0 | 0 |
|  | Day of Life | *Klebsiella quasivariicola* | 0.586 | 0.11106 | 0 | 0 |
|  | Day of Life | *Klebsiella variicola* | 0.72726 | 0.15678 | 0 | 9.00E-05 |
|  | Day of Life | *Lachnoclostridium phytofermentans* | 0.61766 | 0.07372 | 0 | 0 |
|  | Day of Life | *Morganella morganii* | 1.4862 | 0.15896 | 0 | 0 |
|  | Day of Life | *Paeniclostridium sordellii* | 0.81659 | 0.1027 | 0 | 0 |
|  | Day of Life | *Paraclostridium bifermentans* | 0.81758 | 0.08569 | 0 | 0 |
|  | Day of Life | *Providencia sneebia* | 0.67037 | 0.08163 | 0 | 0 |
|  | Day of Life | *Pseudomonas aeruginosa* | 0.40304 | 0.11857 | 0.00073 | 0.00829 |
|  | Day of Life | *Raoultella ornithinolytica* | 0.54456 | 0.08891 | 0 | 0 |
|  | Day of Life | *Romboutsia hominis* | 0.80067 | 0.08869 | 0 | 0 |
|  | Day of Life | *Romboutsia ilealis* | 0.78904 | 0.08651 | 0 | 0 |
|  | Day of Life | *Romboutsia sp CE17* | 0.80055 | 0.0898 | 0 | 0 |
|  | Day of Life | *Rothia mucilaginosa* | 0.76998 | 0.09608 | 0 | 0 |
|  | Day of Life | *Ruminococcus gnavus* | 0.77821 | 0.08619 | 0 | 0 |
|  | Day of Life | *Salmonella enterica* | 0.77534 | 0.1314 | 0 | 0 |
|  | Day of Life | *Shigella boydii* | 0.71545 | 0.09877 | 0 | 0 |
|  | Day of Life | *Shigella dysenteriae* | 0.55378 | 0.08614 | 0 | 0 |
|  | Day of Life | *Shigella flexneri* | 1.01659 | 0.13452 | 0 | 0 |
|  | Day of Life | *Shigella sonnei* | 0.51497 | 0.07956 | 0 | 0 |
|  | Day of Life | *Staphylococcus epidermidis* | -0.84201 | 0.20864 | 6.00E-05 | 0.00099 |
|  | Day of Life | *Streptococcus dysgalactiae* | 0.57059 | 0.09409 | 0 | 0 |
|  | Day of Life | *Streptococcus mitis* | 0.69234 | 0.09821 | 0 | 0 |
|  | Day of Life | *Streptococcus thermophilus* | 0.79942 | 0.10606 | 0 | 0 |
|  | Day of Life | *Veillonella atypica* | 0.82972 | 0.12817 | 0 | 0 |
|  | Day of Life | *Veillonella dispar* | 0.81448 | 0.11884 | 0 | 0 |
|  | Day of Life | *Veillonella parvula* | 1.35198 | 0.15802 | 0 | 0 |
|  | Day of Life | *Veillonella rodentium* | 0.46637 | 0.0816 | 0 | 0 |
|  | Day of Life | *Veillonella sp T1 7* | 0.85909 | 0.11105 | 0 | 0 |
|  | Gestational Age | *Cutibacterium acnes* | 1.23249 | 0.34085 | 0.00059 | 0.0068 |
|  | Probiotic | *Amedibacterium intestinale* | -0.97257 | 0.23991 | 6.00E-05 | 0.00094 |
|  | Probiotic | *Arcobacter cryaerophilus* | -0.97679 | 0.24056 | 6.00E-05 | 0.00092 |
|  | Probiotic | *Bacillus cereus* | -0.9396 | 0.27322 | 0.00063 | 0.00728 |
|  | Probiotic | *Bacillus megaterium* | -0.98977 | 0.24502 | 6.00E-05 | 0.00099 |
|  | Probiotic | *Bacillus mycoides* | -0.81609 | 0.22956 | 0.00041 | 0.00505 |
|  | Probiotic | *Bifidobacterium bifidum* | 1.60971 | 0.28277 | 0 | 0 |
|  | Probiotic | *Bifidobacterium breve* | 4.91735 | 0.61814 | 0 | 0 |
|  | Probiotic | *Bifidobacterium longum* | 3.26837 | 0.49296 | 0 | 0 |
|  | Probiotic | *Caloramator sp E03* | -1.01815 | 0.25399 | 7.00E-05 | 0.0011 |
|  | Probiotic | *Caprobacter fermentans* | -0.96217 | 0.25468 | 0.00018 | 0.00246 |
|  | Probiotic | *Cellulosilyticum sp WCF 2* | -0.89549 | 0.2449 | 0.00028 | 0.00361 |
|  | Probiotic | *Citrobacter freundii* | 1.09921 | 0.37619 | 0.00363 | 0.0365 |
|  | Probiotic | *Clostridioides difficile* | -1.92875 | 0.45874 | 3.00E-05 | 0.00055 |
|  | Probiotic | *Clostridium acetobutylicum* | -1.00675 | 0.28475 | 0.00044 | 0.0054 |
|  | Probiotic | *Clostridium argentinense* | -1.17761 | 0.30849 | 0.00015 | 0.00215 |
|  | Probiotic | *Clostridium autoethanogenum* | -0.81514 | 0.22356 | 0.00029 | 0.00374 |
|  | Probiotic | *Clostridium baratii* | -1.44092 | 0.41596 | 0.00058 | 0.00671 |
|  | Probiotic | *Clostridium beijerinckii* | -2.00245 | 0.47607 | 3.00E-05 | 0.00055 |
|  | Probiotic | *Clostridium bornimense* | -1.32541 | 0.36213 | 0.00028 | 0.00358 |
|  | Probiotic | *Clostridium botulinum* | -1.97143 | 0.47848 | 4.00E-05 | 0.00074 |
|  | Probiotic | *Clostridium butyricum* | -2.16306 | 0.57886 | 0.00021 | 0.00279 |
|  | Probiotic | *Clostridium cadaveris* | -1.28865 | 0.3416 | 0.00018 | 0.0025 |
|  | Probiotic | *Clostridium carboxidivorans* | -1.16188 | 0.26945 | 2.00E-05 | 0.00036 |
|  | Probiotic | *Clostridium cellulovorans* | -1.13954 | 0.29316 | 0.00011 | 0.0017 |
|  | Probiotic | *Clostridium chauvoei* | -1.11002 | 0.35795 | 0.00203 | 0.02123 |
|  | Probiotic | *Clostridium cochlearium* | -1.01517 | 0.26059 | 0.00011 | 0.00165 |
|  | Probiotic | *Clostridium diolis* | -1.31548 | 0.3461 | 0.00016 | 0.00226 |
|  | Probiotic | *Clostridium drakei* | -0.86955 | 0.2465 | 0.00046 | 0.00553 |
|  | Probiotic | *Clostridium estertheticum* | -1.1342 | 0.29467 | 0.00013 | 0.00193 |
|  | Probiotic | *Clostridium formicaceticum* | -1.01685 | 0.24268 | 3.00E-05 | 0.00058 |
|  | Probiotic | *Clostridium gasigenes* | -1.26739 | 0.35587 | 4.00E-04 | 0.00496 |
|  | Probiotic | *Clostridium innocuum* | -1.38055 | 0.30227 | 1.00E-05 | 0.00012 |
|  | Probiotic | *Clostridium intestinale* | -1.22669 | 0.34052 | 0.00035 | 0.00432 |
|  | Probiotic | *Clostridium isatidis* | -1.17323 | 0.35202 | 0.00092 | 0.01038 |
|  | Probiotic | *Clostridium kluyveri* | -1.27871 | 0.31236 | 5.00E-05 | 0.00082 |
|  | Probiotic | *Clostridium ljungdahlii* | -0.98922 | 0.28082 | 0.00046 | 0.00558 |
|  | Probiotic | *Clostridium novyi* | -1.17037 | 0.30253 | 0.00012 | 0.00181 |
|  | Probiotic | *Clostridium pasteurianum* | -1.22125 | 0.31874 | 0.00014 | 0.00205 |
|  | Probiotic | *Clostridium saccharobutylicum* | -2.09638 | 0.45517 | 1.00E-05 | 1.00E-04 |
|  | Probiotic | *Clostridium saccharoperbutylacetonicum* | -1.90354 | 0.4426 | 2.00E-05 | 0.00037 |
|  | Probiotic | *Clostridium scatologenes* | -0.89778 | 0.24499 | 0.00027 | 0.00355 |
|  | Probiotic | *Clostridium scindens* | -1.00392 | 0.27391 | 0.00027 | 0.00355 |
|  | Probiotic | *Clostridium septicum* | -1.28438 | 0.36856 | 0.00053 | 0.00631 |
|  | Probiotic | *Clostridium sp AWRP* | -0.96321 | 0.26186 | 0.00026 | 0.00343 |
|  | Probiotic | *Clostridium sp C5S11* | -2.0173 | 0.44358 | 1.00E-05 | 0.00013 |
|  | Probiotic | *Clostridium sp CT4* | -1.36533 | 0.34808 | 1.00E-04 | 0.0015 |
|  | Probiotic | *Clostridium sp DL VIII* | -1.77244 | 0.42871 | 4.00E-05 | 0.00072 |
|  | Probiotic | *Clostridium sp JN 1* | -1.01704 | 0.26391 | 0.00013 | 0.00191 |
|  | Probiotic | *Clostridium sp JN 9* | -1.00468 | 0.2577 | 0.00011 | 0.00164 |
|  | Probiotic | *Clostridium sp JN500901* | -0.96794 | 0.25393 | 0.00015 | 0.00218 |
|  | Probiotic | *Clostridium sp M62 1* | -0.98125 | 0.24428 | 7.00E-05 | 0.00107 |
|  | Probiotic | *Clostridium sp MF28* | -1.52413 | 0.41965 | 0.00031 | 0.00391 |
|  | Probiotic | *Clostridium sp SYSU GA15002* | -1.01308 | 0.25804 | 1.00E-04 | 0.00149 |
|  | Probiotic | *Clostridium sporogenes* | -1.4002 | 0.426 | 0.00108 | 0.01196 |
|  | Probiotic | *Clostridium taeniosporum* | -1.56511 | 0.42586 | 0.00026 | 0.00346 |
|  | Probiotic | *Clostridium tetani* | -1.08261 | 0.28116 | 0.00013 | 0.00193 |
|  | Probiotic | *Clostridium tyrobutyricum* | -1.11096 | 0.27361 | 6.00E-05 | 0.00092 |
|  | Probiotic | *Enterobacter cancerogenus* | 0.68382 | 0.25091 | 0.00664 | 0.06091 |
|  | Probiotic | *Enterobacter cloacae* | 1.52373 | 0.41104 | 0.00023 | 0.00312 |
|  | Probiotic | *Enterobacter roggenkampii* | 1.20025 | 0.4228 | 0.00471 | 0.04656 |
|  | Probiotic | *Finegoldia magna* | -1.17891 | 0.38005 | 0.00203 | 0.02123 |
|  | Probiotic | *Fusobacterium nucleatum* | -1.46027 | 0.26907 | 0 | 0 |
|  | Probiotic | *Hathewaya histolytica* | -0.88514 | 0.24327 | 3.00E-04 | 0.00383 |
|  | Probiotic | *Klebsiella aerogenes* | 1.16902 | 0.45259 | 0.01007 | 0.08086 |
|  | Probiotic | *Klebsiella grimontii* | 0.8828 | 0.32454 | 0.00674 | 0.06154 |
|  | Probiotic | *Klebsiella michiganensis* | 1.38382 | 0.40466 | 0.00068 | 0.00776 |
|  | Probiotic | *Klebsiella oxytoca* | 1.18487 | 0.41111 | 0.00411 | 0.041 |
|  | Probiotic | *Klebsiella quasipneumoniae* | 1.52542 | 0.48974 | 0.00194 | 0.02046 |
|  | Probiotic | *Lachnoclostridium phytofermentans* | -0.92722 | 0.22622 | 5.00E-05 | 0.00081 |
|  | Probiotic | *Paeniclostridium sordellii* | -1.12961 | 0.31501 | 0.00037 | 0.00457 |
|  | Probiotic | *Paraclostridium bifermentans* | -1.0026 | 0.26266 | 0.00015 | 0.00215 |
|  | Probiotic | *Providencia sneebia* | -0.92633 | 0.25051 | 0.00024 | 0.0032 |
|  | Probiotic | *Raoultella ornithinolytica* | 0.83159 | 0.27249 | 0.00239 | 0.02447 |
|  | Probiotic | *Romboutsia hominis* | -1.07452 | 0.27187 | 9.00E-05 | 0.00136 |
|  | Probiotic | *Romboutsia ilealis* | -0.95517 | 0.26514 | 0.00035 | 0.00432 |
|  | Probiotic | *Romboutsia sp CE17* | -1.10256 | 0.27528 | 7.00E-05 | 0.0011 |
|  | Probiotic | *Rothia mucilaginosa* | -1.40962 | 0.29495 | 0 | 5.00E-05 |
|  | Probiotic | *Ruminococcus gnavus* | -0.91886 | 0.26452 | 0.00056 | 0.00654 |
|  | Probiotic | *Salmonella enterica* | 1.31679 | 0.40356 | 0.00117 | 0.01293 |
|  | Probiotic | *Staphylococcus epidermidis* | 1.75765 | 0.64204 | 0.00639 | 0.05959 |
|  | Probiotic | *Streptococcus agalactiae* | -1.9903 | 0.40949 | 0 | 3.00E-05 |
|  | Probiotic | *Streptococcus dysgalactiae* | -1.64324 | 0.28895 | 0 | 0 |
|  | Probiotic | *Streptococcus mitis* | -1.39087 | 0.30138 | 0 | 1.00E-04 |
|  | Race | *Clostridium botulinum* | 1.81932 | 0.70646 | 0.01233 | 0.09597 |
|  | Race | *Clostridium sporogenes* | 1.71858 | 0.60595 | 0.0061 | 0.05795 |
|  | Sex | *Rothia mucilaginosa* | 0.80248 | 0.28778 | 0.00694 | 0.06294 |
| **Enteral Nutrition** | |  |  |  |  |  |
|  | EBM | *Citrobacter sp Y3* | -0.62284 | 0.22053 | 0.00493 | 0.04752 |
|  | EBM | *Paeniclostridium sordellii* | -0.9139 | 0.35894 | 0.01118 | 0.08782 |
|  | EBM | *Streptococcus agalactiae* | -1.98783 | 0.46677 | 2.00E-05 | 0.00044 |
|  | Formula | *Paeniclostridium sordellii* | -0.67593 | 0.18893 | 0.00038 | 0.00468 |
|  | Formula | *Streptococcus agalactiae* | -0.86133 | 0.24612 | 0.00051 | 0.00604 |
|  | PDHM | *Citrobacter koseri* | -1.08414 | 0.36292 | 0.00295 | 0.02991 |
|  | PDHM | *Citrobacter sp Y3* | -0.58106 | 0.20589 | 0.00495 | 0.0476 |
|  | PDHM | *Paeniclostridium sordellii* | -1.15678 | 0.33339 | 0.00056 | 0.00659 |
|  | PDHM | *Streptococcus agalactiae* | -2.01397 | 0.43435 | 0 | 9.00E-05 |

| **Table S2.** Multilocus sequence typing of *Streptococcus agalactiae* BSI isolates | | | | | | | | | |
| --- | --- | --- | --- | --- | --- | --- | --- | --- | --- |
| **Patient** | **Loci** | | | | | | | **Sequence Type** | **Clonal Complex** |
|  | **adhP** | **atr** | **glcK** | **glnA** | **pheS** | **sdhA** | **tkt** |  |  |
| AD8134 | 2 | 1 | 1 | 2 | 1 | 1 | 1 | ST17 | cc17 |
| PL2596 | 5 | 4 | 2 | 2 | 4 | 2 | 3 | ST24 | cc452 |
| QA9843 | 2 | 1 | 1 | 2 | 1 | 1 | 1 | ST17 | cc17 |
| YH3562 | 5 | 4 | 2 | 2 | 4 | 2 | 3 | ST24 | cc452 |
| ZR1676 | 5 | 4 | 2 | 2 | 4 | 2 | 3 | ST24 | cc452 |
| BSI, bloodstream infection; adhP, alcohol dehydrogenase; atr, glutamine transporter protein; glcK, glucose kinase; glnA, glutamine synthetase; pheS, phenylalanyl tRNA synthetase; sdhA, serine dehydratase; tkt, transketolase | | | | | | | | | |

| **Table S3.** Statistics for genomes generated from BSI isolates | | | | |
| --- | --- | --- | --- | --- |
| **Genome** | **Completeness** | **Contamination** | **Strain heterogeneity** | **Estimated coverage** |
| AD8134_BC0 | 100.00 | 0.00 | 0.00 | 1531.3 |
| AT4275_BC9 | 99.65 | 0.09 | 0.00 | 1228.3 |
| HJ2545_BC20 | 100.00 | 0.00 | 0.00 | 98.6 |
| JU9212_BC53 | 99.51 | 0.08 | 0.00 | 99.1 |
| NR7313_BC4 | 99.81 | 0.19 | 0.00 | 797.0 |
| NZ7258_BC15 | 100.00 | 0.91 | 0.00 | 519.3 |
| PL2596_BC65 | 100.00 | 0.00 | 0.00 | 1067.8 |
| QA9843_BC32 | 100.00 | 0.00 | 0.00 | 1314.4 |
| RN6899_BC10 | 99.70 | 0.33 | 0.00 | 750.4 |
| RX6831_BC24 | 100.00 | 0.07 | 0.00 | 117.3 |
| RX8994_BC14 | 99.97 | 0.52 | 0.00 | 123.8 |
| SG3906_BC50 | 99.81 | 0.00 | 0.00 | 1188.5 |
| TN3774_BC6 | 99.97 | 0.24 | 0.00 | 709.4 |
| YC2596_BC26 | 99.51 | 0.08 | 0.00 | 1107.6 |
| YH3562_BC0 | 100.00 | 0.00 | 0.00 | 433.4 |
| ZR1676_BC35 | 100.00 | 0.00 | 0.00 | 1028.7 |
